# Identification and subsequent validation of transcriptomic signature associated with metabolic status in endometrial cancer

**DOI:** 10.1038/s41598-023-40994-w

**Published:** 2023-08-23

**Authors:** Iwona Sidorkiewicz, Maciej Jóźwik, Angelika Buczyńska, Anna Erol, Marcin Jóźwik, Marcin Moniuszko, Katarzyna Jarząbek, Magdalena Niemira, Adam Krętowski

**Affiliations:** 1 Clinical Research Centre, Medical University of Białystok, Marii Skłodowskiej-Curie 24a, 15-276 Białystok, Poland; 2 Department of Gynecology and Gynecologic Oncology, Medical University of Białystok, 15-276 Białystok, Poland; 3 Department of Gynecology and Obstetrics, Collegium Medicum, University of Warmia and Mazury in Olsztyn, 10-045 Olsztyn, Poland; 4 Department of Regenerative Medicine and Immune Regulation, Medical University of Bialystok, 15-269 Białystok, Poland; 5 Department of Allergology and Internal Medicine, Medical University of Bialystok, 15-276 Białystok, Poland; 6 Laboratory of Genetic and Molecular Diagnostics, Maria Skłodowska-Curie Białystok Oncology Center, 15-027 Białystok, Poland; 7 Department of Endocrinology, Diabetology and Internal Medicine, Medical University of Białystok, 15-276 Białystok, Poland

**Keywords:** Cancer genomics, Cancer metabolism, Transcriptomics, Cancer metabolism

## Abstract

Aberrant metabolism has been identified as a main driver of cancer. Profiling of metabolism-related pathways in cancer furthers the understanding of tumor plasticity and identification of potential metabolic vulnerabilities. In this prospective controlled study, we established transcriptomic profiles of metabolism-related pathways in endometrial cancer (EC) using a novel method, NanoString nCounter Technology. Fifty-seven ECs and 30 normal endometrial specimens were studied using the NanoString Metabolic Panel, further validated by qRT-PCR with a very high similarity. Statistical analyses were by GraphPad PRISM and Weka software. The analysis identified 11 deregulated genes (FDR ≤ 0.05; |FC|≥ 1.5) in EC: *SLC7A11*; *SLC7A5*; *RUNX1*; *LAMA4*; *COL6A3*; *PDK1*; *CCNA1*; *ENO1*; *PKM*; *NR2F1*; and *NAALAD2*. Gene ontology showed direct association of these genes with ‘central carbon metabolism (CCM) in cancer’. Thus, ‘CCM in cancer’ appears to create one of the main metabolic axes in EC. Further, transcriptomic data were functionally validated with drug repurposing on three EC cell lines, with several drug candidates suggested. These results lay the foundation for personalized therapeutic strategies in this cancer. Metabolic plasticity represents a promising diagnostic and therapeutic option in EC.

## Introduction

Endometrial cancer (EC) remains one of the most common cancers of the female reproductive system and its incidence is expected to rise sharply over the next decades^1^. Epidemiological and clinical evidence implicates metabolic abnormalities in its development^2^. However, the etiology of this disease is still debated. A categorization of EC involves two clinical types: Type I which encompasses endometrioid grade 1 (well differentiated) and grade 2 (moderately differentiated) tumors, and Type II: undifferentiated grade 3 of any histotype. Type I represents the vast majority (80–90%) of cases, especially in obese pre-, peri-, and early post-menopausal women, whereas Type II occurs mostly in postmenopausal women and has a high risk of relapse and metastatic disease^3^. Moreover, a new classification of EC by The Cancer Genome Atlas Consortium has been implemented, which considers four molecular subtypes. Molecular subtyping may yield information on cancer biology, prognosis and treatment benefits; however, a long-term follow-up is needed for the development of new management approaches^4,5^.

Metabolic reprogramming has been identified as one of the main drivers of cancer. Recent literature has established a molecular link between oncogenic pathways and tumor metabolism^6^. Cancer cells have an increased energy uptake and utilization compared with normal cells^7^ and prefer to convert glucose to pyruvate and lactate by glycolysis even in the presence of an adequate oxygen supply. This deviant energetic metabolism, known as the Warburg effect, is characterized by a very high rate of anerobic glycolysis and an uptake and incorporation of resultant nutrients for increased biosynthesis alternatively to efficient adenosine triphosphate (ATP) production^8^. Metabolic profiling in EC showed increased rates of glycolysis and decreased glucose oxidation than in normal endometrial cells without the reduction of oxidative phosphorylation (OXPHOS)^9^. The role of the Warburg effect in cancer pathogenesis has yet to be established, however, being a therapeutic vulnerability, such an altered metabolism may become a window for new therapeutics in EC patients^10^.

Transcriptomics has the potential to identify particular metabolic phenotypes that are associated with cancer and provide insights into the mechanistic pathways involved in cancer development and progression. To date, there has been no study specifically looking at the transcriptomic analysis of metabolism in EC. Therefore, the broad goal of this investigation was to establish transcriptomic signature in EC to comprehensively characterize its metabolic status and provide gene expression fingerprint. Pathways and network connections were verified to further explore the relationships between the differentially expressed genes (DEGs). Such an approach could give a considerable background for the clinical management of EC patients and provide an important tool for future research. Consequently, the results of this study, verified on three separate cell lines, highlight the new molecular features of EC, and provide insight into the events underlying the development and progression of EC.

## Results

### Metabolism-related transcriptomic changes in endometrial cancer and functional enrichment analysis

**Figure 1:**
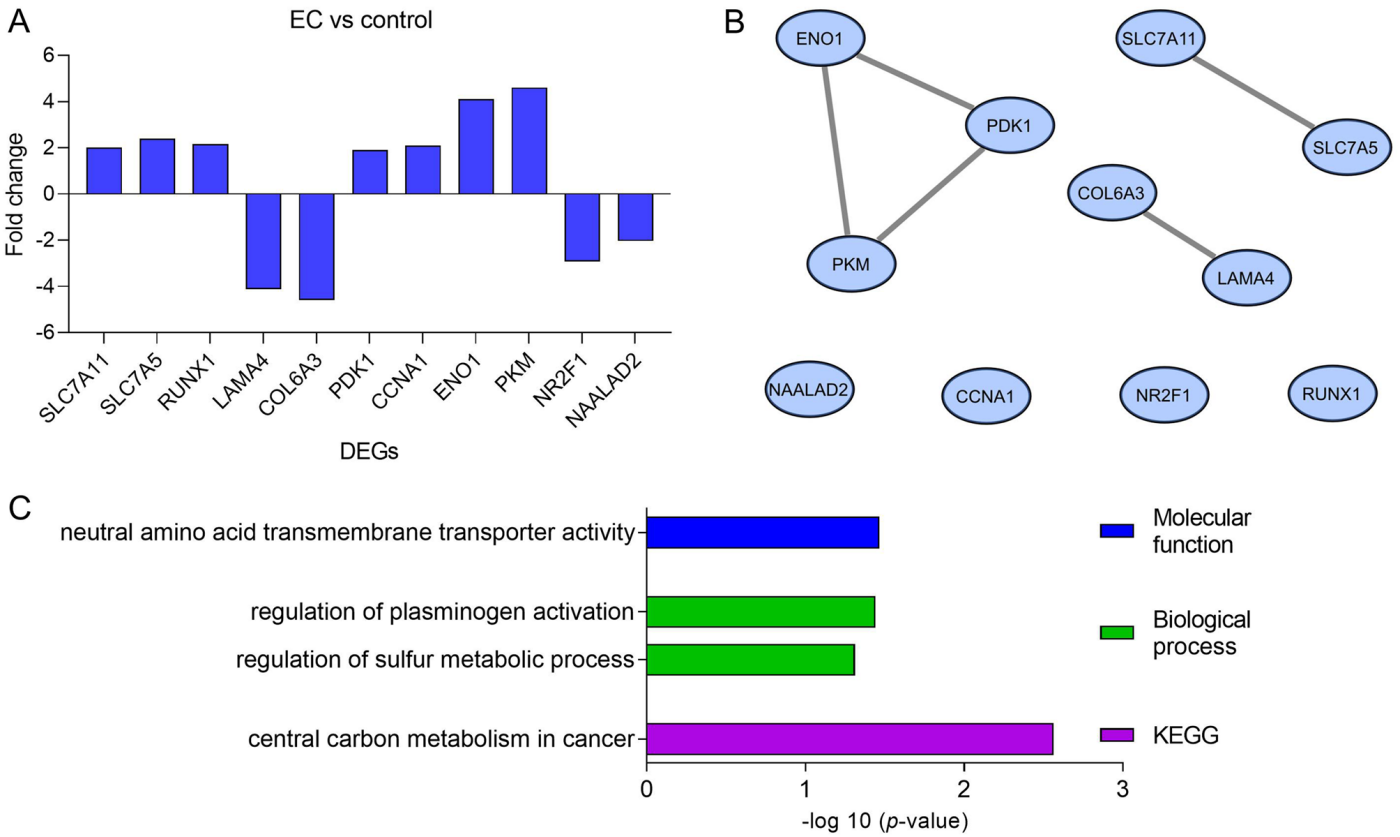
DEGs in EC. (**A**) DEGs with significantly different expressions between the EC group (N = 57) and reference group (N = 30) (FDR ≤ 0.05; |FC| ≥ 1.5). (**B**) The DEGs network rendered using the STRING database. (**C**) Significantly enriched GO (− log_10_(*p*-value)) categories of the DEGs in the molecular function, biological process, and KEGG enrichment. DEGs, differentially expressed genes; EC, endometrial cancer; FC, fold change; FDR, false discovery rate; GO, Gene Ontology; KEGG, Kyoto Encyclopedia of Genes and Genomes.

**Figure 2:**
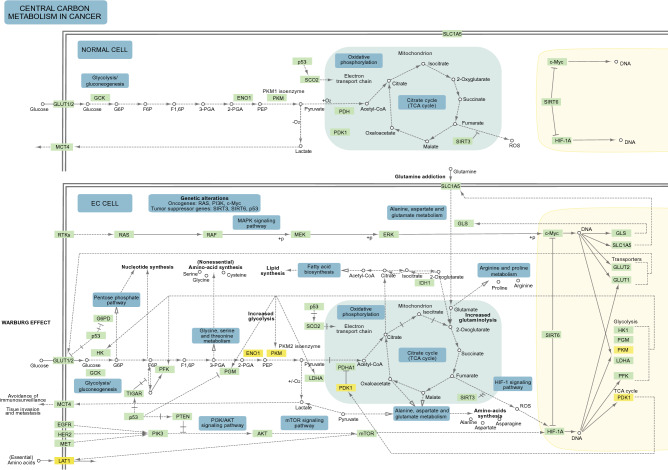
Central carbon metabolism in cancer. Adapted from the KEGG using KEGGParser plugin and Cytoscape App.

The metabolic profiling of 768 genes was performed using the NanoString Technology platform. Eleven genes were identified as differentially expressed between EC and controls based on a false discovery rate (FDR) threshold of ≤ 0.05 and |fold change (FC)| of ≥ 1.5 (Fig. 1A). All the DEGs were entered into the Search Tool for Retrieval of Interacting Genes/Proteins (STRING) database to obtain the interaction data. After analysis, 11 nodes were shown to be interacting with five edges having a 0.909 average node degree and 0.636 average local clustering coefficient. The protein–protein interaction (PPI) enrichment value was predicted to be 0.00445 (Fig. 1B). Gene ontology (GO) was applied for identifying and visualizing the appropriate biological pathways and processes associated with the DEGs. The GO analysis of the EC network biological process revealed a predominant role of the following categories: ‘regulation of plasminogen activation’ and ‘regulation of sulfur metabolic process’. In terms of GO molecular function annotations, these genes were involved in ‘neutral amino acid transmembrane transporter activity’. Additionally, the Kyoto Encyclopedia of Genes and Genomes (KEGG) pathway enrichment analysis confirmed that ‘central carbon metabolism (CCM) in cancer’ is involved in the EC metabolism (Figs. 1C and 2).

Likewise, gene signatures in EC histologic types were identified based on an FDR threshold of ≤ 0.05 and |FC| of ≥ 1.5 (Figs. S1 and S2).

### Data validation

**Figure 3:**
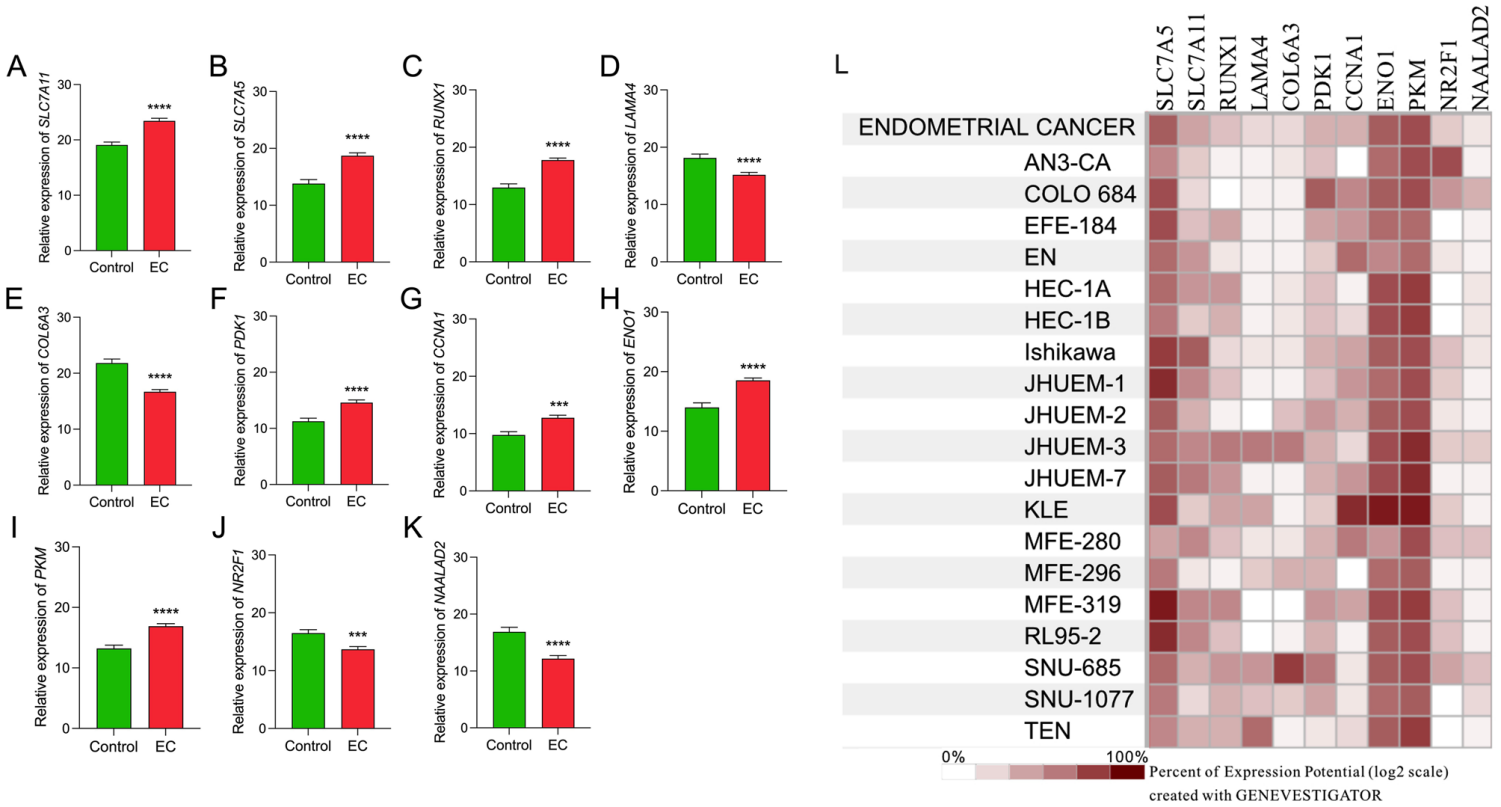
Relative mRNA expression of DEGs in EC (N = 57) and reference endometrial tissue (N = 30). (**A**) *SLC7A11*; (**B**) *SLC7A5*; (**C**) *RUNX1*; (**D**) *LAMA4*; (**E**) *COL6A3*; (**F**) *PDK1*; (**G**) *CCNA1*; (**H**) *ENO1*; (**I**) *PKM*; (**J**) *NR2F1*; (**K**) *NAALAD2*. Each bar represents the geometric mean ± standard error of mean (SEM). Gene expression was normalized on both peptidylprolyl isomerase A and beta-actin housekeeping genes. Levels of significance difference (Mann–Whitney U-test) when compared with control: ***p < 0.001; ****p < 0.0001; (**L**) Validation of the discovered DEGs in EC cell lines by Genevestigator software (v.9.10.0).

The DEGs from the EC metabolic profiling were validated to further verify the results of the NanoString analysis. A total of 87 formalin-fixed paraffin-embedded (FFPE) samples from the study and reference groups were investigated using quantitative Real-Time PCR (qRT-PCR) for the relative expression levels of the 11 discovered DEGs (Fig. 3A–K). The qRT-PCR validation results demonstrated a very high similarity when compared with the expression profiles established by the NanoString, thus confirming the importance of all 11 DEGs. Moreover, the expression pattern of DEGs in EC cell lines was confirmed with Genevestigator software (Fig. 3L).

### Diagnostic value

The diagnostic value of the DEGs as candidate EC biomarkers was evaluated by the area under the receiver operating characteristic curve (AUC). The highest AUC and, hence, possible clinical applicability as EC diagnostic marker, was observed for solute carrier family 7 member 5 (*SLC7A5)* (AUC = 0.67) gene expression (Table 1). Moreover, using a logistic regression model, we screened all the 768 genes for constructing an EC diagnostic signature. Logistic regression selected two candidate genes: C-X-C motif chemokine ligand 9 (*CXCL9*) and 3-hydroxyanthranilate 3,4-dioxygenase (*HAAO*). Consequently, a model for comparison of EC vs control showed their higher diagnostic value (AUC = 0.79; Table 2) compared to the highest AUC found for any separate DEG.

**Table 1:** The highest values of AUC found for metabolism-related DEGs.

Gene	Full name	FC	FDR	AUC	95%CI
*SLC7A5*	Solute carrier family 7 member 5	2.39	0.00	0.67	0.556–0.776
*PKM*	Pyruvate kinase M1/2	4.61	0.04	0.65	0.538–0.764
*ENO1*	Enolase 1	4.10	0.04	0.64	0.525–0.754

AUC, area under the receiver operating characteristic curve; CI, confidence intervals; EC, endometrial cancer; FC, fold change; FDR, false discovery rate.

**Table 2:** The basic parameters and common quality measures of the proposed model distinguishing EC from control samples in logistic regression.

Gene expression	Odds ratio	TP rate	FP rate	Precision	ROC area	Intercept	Coefficient
x1 = *CXCL9*	0.06	0.770	0.326	0.764	0.793	− 1.1113	a1 = − 2.8277
x 2 = *HAAO*	21.76						a2 = 3.0802

*CXCL9*, C-X-C motif chemokine ligand 9; *HAAO*, 3-hydroxyanthranilate 3,4-dioxygenase; FP, false positive; ROC, receiver operating characteristic; TP, true positive.

The confusion matrix of the naive Bayes model for comparison of EC vs control was further performed. This model correctly classified 15 out of 30 controls. In addition, one of the tumor samples was incorrectly recognized. Interestingly, the J48 modeling for distinguishing EC from controls showed a comparable result: the model incorrectly categorizing 14 controls and 13 EC samples.

### In silico identification of drugs for the treatment of EC

Through the exploration of four dedicated gene-drug interaction databases, a total of five drugs related to the 11 DEGs based on their possible effect on cell viability and metabolic signature in EC were selected. These drugs were found to be mainly related to four DEGs: RUNX family transcription factor 1 (*RUNX1)*, *SLC7A5*, solute carrier family 7 member 11 (*SLC7A11)*, and pyruvate kinase M1/2 (*PKM)* (Table 3). 17β-Estradiol was used to study the estrogen-associated regulation of metabolism in EC^11,12^. In addition, cisplatin, a well-known chemotherapeutic drug that acts by crosslinking with the nitrogen of guanine in DNA, was used to compare the effects of repurposed drugs and standard treatment.

**Table 3:** Candidate drugs for EC treatment.

Repurposed drug (DrugBank ID)	Target gene(s)	Brief description
Sapanisertib (INK-128) (DB11836)	*SLC7A5*, *RUNX1*, *ENO1*, *LAMA4*	An investigational, oral, highly selective ATP-competitive mTOR kinase inhibitor
PHA-793887 (DB12686)	*SLC7A5*, *ENO1*, *NR2F1*, *COL6A3*	A novel and potent inhibitor of multiple cyclin dependent kinases (CDK) with a particular activity against CDK2, − 5, and − 7
Ciclopirox (DB01188)	*PKM*, *SLC7A11*, *PDK1*	Broad-spectrum topical antifungal agent used to treat mild to moderate onychomycosis of fingernails and toenails
Sulfasalazine (DB00795)	*SLC7A11*	Nonsteroidal anti-inflammatory drug used to treat Crohn's disease and rheumatoid arthritis
Cytarabine (DB00987)	*RUNX1*	Pyrimidine nucleoside analogue used to treat acute non-lymphocytic leukemia, lymphocytic leukemia, and the blast phase of chronic myelocytic leukemia

### Functional validation

**Figure 4:**
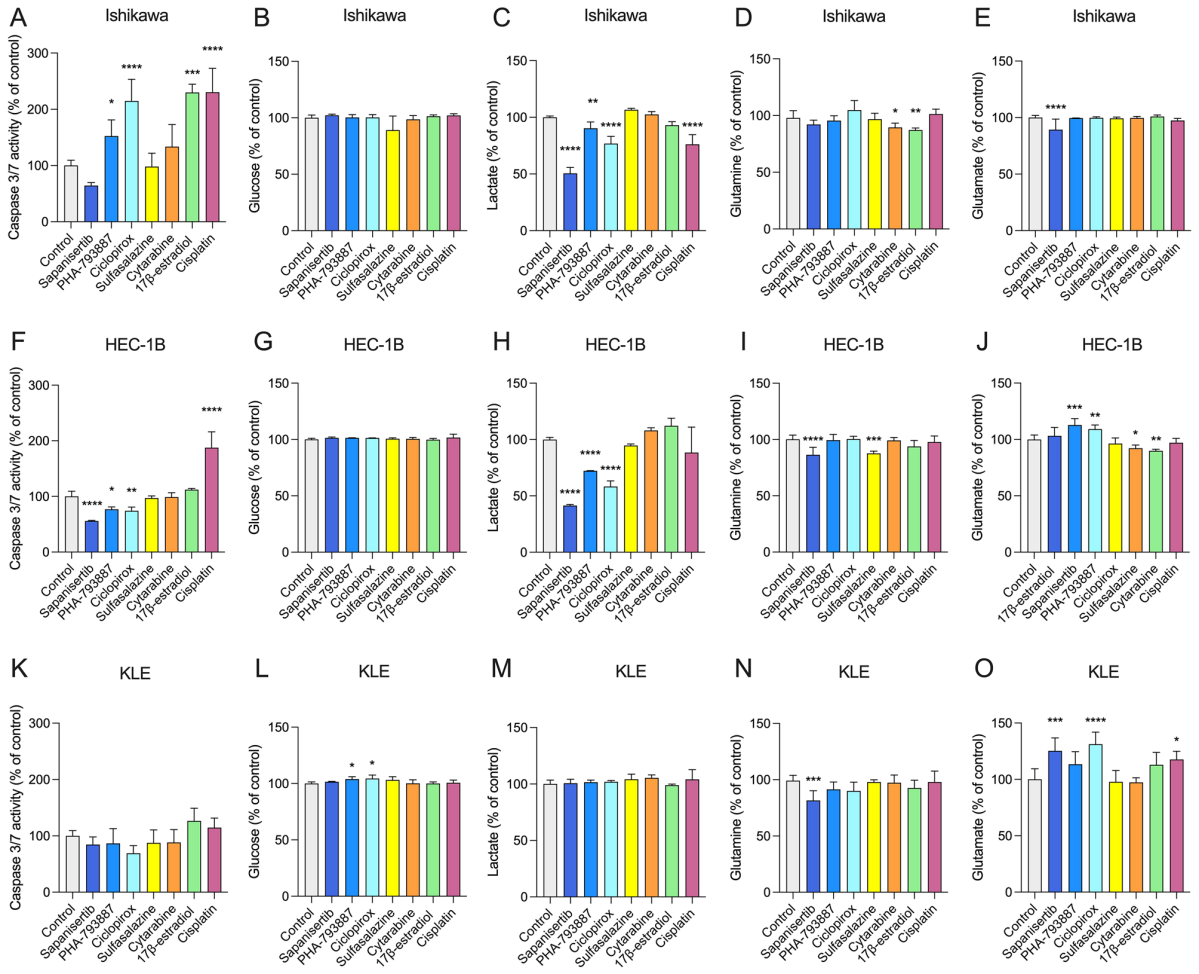
The effects of sapanisertib (10^−7^ M); PHA-793887 (10^−6^ M); ciclopirox (10^−6^ M); sulfasalazine (10^−6^ M); cytarabine (10^−5^ M); 17β-estradiol (10^−5^ M); and cisplatin (10^−5^ M) on caspase 3/7 activity and cellular metabolism in EC cells. The Ishikawa, HEC-1B, and KLE cells were treated with drugs for 48 h as described in methods. (**A**), (**F**), (**K**)—Caspase 3/7 activity. (**B**), (**G**), (**L**)—Extracellular glucose concentration. (**C**), (**H**), (**M**)—extracellular lactate concentration. (**D**), (**I**), (**N**)—extracellular glutamine concentration. (**E**), (**J**), (**O**)—extracellular glutamate concentration. (**A**)–(**E**)—Ishikawa cells. (**F**)–(**J**)—HEC-1B cells. (**K**)–(**O**)—KLE cells. Data are shown as the mean ± SEM of three independent experiments run in triplicates. Levels of significance difference (Mann–Whitney U-test) when compared with control: *p < 0.05; **p  < 0.01; ***p < 0.001; ****p < 0.0001.

Three EC cell lines: Ishikawa, HEC-1B, and KLE were selected to in vitro validate the drug repurposing results (Fig. 3L). All studied compounds decreased EC cell viability at a concentration range (10^−9^ M–10^−5^ M) (Fig. S3). Based on preliminary viability assays, specific concentrations of drugs were used: sapanisertib: 10^−7^ M; PHA-793887: 10^−6^ M; ciclopirox: 10^−6^ M; sulfasalazine: 10^−6^ M; cytarabine: 10^−5^ M; 17β-estradiol: 10^−5^ M; and cisplatin: 10^−5^ M. These experiments demonstrated that 48-h treatment with PHA-793887 (*p* < 0.05), ciclopirox (*p* < 0.05), 17β-estradiol (*p* < 0.0001), and cisplatin (*p* < 0.0001) increased caspase 3/7 activity in Ishikawa cells. Moreover, only cisplatin increased caspase 3/7 activity in HEC-1B cells (*p* < 0.0001). However, sapanisertib (*p* < 0.0001), PHA-793887 (*p* < 0.05), and ciclopirox (*p* < 0.01) decreased caspase 3/7 activity in HEC-1B cells, suggesting other cytotoxic mechanism(s) of their action in this particular EC model. None of the studied compounds affected caspase 3/7 activity in KLE cells (all *p* > 0.05) (Fig. 4A,F,K).

Next, glucose, lactate, glutamine, and glutamate were measured in cell culture medium to describe the metabolite consumption and, therefore, metabolism fluctuations/fluxes during potential therapeutic treatment. Interestingly, only PHA-793887 and ciclopirox reduced glucose consumption in KLE cells (both *p* < 0.05). There were no significant changes in glucose consumption after other drug treatment in any EC cells (*p* > 0.05) (Fig. 4B,G,L). Sapanisertib, PHA-793887 and ciclopirox decreased lactate production in Ishikawa (*p* < 0.0001; *p* < 0.05; *p* < 0.0001; respectively) and HEC-1B (all *p* < 0.0001) cells. Moreover, cisplatin decreased lactate concentration in Ishikawa cell medium (*p* < 0.001) (Fig. 4C,H,M). Decreased extracellular glutamine was observed after cytarabine and 17β-estradiol treatment on Ishikawa cells (*p* < 0.05; *p* < 0.01; respectively), after sapanisertib and sulfasalazine treatment on HEC-1B cells (*p* < 0.001; *p* < 0.0001; respectively), and after sapanisertib treatment on KLE cells (*p* < 0.001) (Fig. 4D,I,N). Decreased glutamate secretion was demonstrated in Ishikawa cells after sapanisertib (*p* < 0.0001) treatment, in HEC-1B after sulfasalazine (*p* < 0.05), and cytarabine (*p* < 0.01) treatment. Moreover, increased glutamate secretion was observed after sapanisertib (*p* < 0.001) and PHA-793887 treatment (*p* < 0.01) in HEC-1B, and after sapanisertib (*p* < 0.001), ciclopirox (*p* < 0.0001), and cisplatin (*p* < 0.05) treatment in KLE cells (Fig. 4E,J,O).

## Discussion

The standard clinical approach in EC treatment remains surgery and/or chemo- and radiotherapy. Despite a generally good prognosis, EC patients may still suffer from treatment failure and recurrences^13^. New insights into the molecular profile could give novel information to better understand the biology of EC. To date, only a few transcriptomic studies have focused on the regulation of cancer metabolism. Specifically, prognostic gene signatures using transcription factors and immune-related mechanisms were studied^14–17^.

Targeting cancer metabolism is an attractive treatment strategy. Metabolic modeling is highly needed to identify the key control points in EC as possible diagnostic and therapeutic targets. In order to find this unique metabolic gene signature, this study performed a NanoString analysis in EC and compared it with normal endometrial tissue. GO and PPI networks were applied to explain the most significant metabolism-related transcriptome profile alterations.

Our study demonstrated a substantial deregulation of 11 genes in the whole EC group compared with normal endometrium. Let us go briefly over their roles and significance. Importantly, increased gene expression of amino acid transporters: *SLC7A5* and *SLC7A11* in EC was confirmed. *SLC7A5* encodes amino acid transporter LAT1 which was shown to correlate with poor disease-free survival in EC patients, suggesting that its inhibition may be an effective therapeutic strategy^18^. LAT1 exports glutamine in exchange for leucine and other essential amino acids which are critical for metabolic activation and cellular function. Leucine is a well-known activator of mechanistic target of rapamycin (mTOR) signaling^19^. In turn, SLC7A11, a cystine/glutamate antiporter conferring specificity for cystine uptake, promotes the synthesis of reduced glutathione to limit oxidative damage and protect cancer cells from apoptosis^20^. SLC7A11 is also involved in cell protection from ferroptosis, an iron-dependent nonapoptotic cell death induced by accumulation of lipid peroxides in the cell membrane^21,22^. SLC7A11 overexpression has been a known risk factor for worse overall survival in several cancers^23^.

RUNX1 has been shown to be upregulated in most cancers compared with normal tissues^24,25^, in agreement with the results of our study. The interplay between RUNX1 and estrogens has been demonstrated and a crucial role of RUNX1 in promotion of metastasis in EC suggested^26,27^. Since RUNX1 is a DNA-binding protein able to indirectly activate target genes without the involvement of an estrogen response element^24^, the upregulation of *RUNX1* in EC observed in this study suggests that additional non-canonical pathways of estrogen signaling stimulation are present in EC. RUNX1 has also been shown to be associated with cell invasion and migration through the regulation of matrix metalloproteases (MMPs)^28^. Moreover, RUNX1 regulates transcription of genes involved in the Wnt signaling which is essential for epithelial-to-mesenchymal transition (EMT)^29,30^.

Interestingly, collagen type VI alpha 3 chain (*COL6A3*) and laminin subunit alpha 4 (*LAMA4*), both associated with extracellular matrix (ECM) remodeling, have shown to be downregulated in EC. The involvement of COL6A3 in serine-type endopeptidase inhibitor activity suggests its anti-cancerous role. Thus, downregulation of such an activity seems to be oncogenic. COL6A3 has been shown to be downregulated in EC tumorigenesis and upregulated in EC metastasis^31^. LAMA4 has also been observed to be downregulated in ovarian cancer cells^32^. Its role in cancer is thought to mediate the attachment, migration, and organisation of cells into tissues by interacting with other ECM components. Moreover, a study by Thyboll et al. demonstrated a key role of LAMA4 in microvessel growth and build-up of endothelial basement membranes^33^. Therefore, it can be hypothesized that low expression of LAMA4 in EC is associated with lesser maturation of endothelia in cancer neoangiogenesis.

Pyruvate dehydrogenase kinase 1 (PDK1) is a key enzyme overexpressed in metabolic reprogramming of many cancers, and is associated with bad prognosis and resistance to therapy^34^. PDK1 regulates hypoxia-induced glucose metabolism by catalyzing the inactivation of the pyruvate dehydrogenase complex^35^. An in vitro study by Wong et al. showed that dichloroacetate, an inhibitor of pyruvate dehydrogenase kinases, is effective in sensitizing most low-to-moderately invasive EC cells to apoptosis, suggestive of its potential role in cancer therapy^36^.

Cyclin A1 (CCNA1), a cell cycle regulatory protein, was initially identified as a compound essential for spermatogenesis and acute myeloid leukemia development. However, accumulating evidence indicates that upregulation of CCNA1 is closely associated with cancer development, progression, and metastasis in several types of tumors, including EC^37–39^. This cyclin has been shown to bind to crucial cell cycle regulators, such as retinoblastoma family proteins, transcription factor E2F-1, and the p21 family proteins. Moreover, it has been demonstrated that CCNA1 associates with androgen receptor and regulates MMPs and vascular endothelial growth factor expression^40,41^.

In turn, enolase 1 (*ENO1*) is an important gene in anaerobic glycolysis catalyzing its penultimate step. A recent study has shown that *ENO1* is a bifunctional gene that encodes both a glycolytic protein and a c-Myc-binding protein, which support its significance in different tumors^42^. Moreover, Song et al. demonstrated that *ENO1* silencing shifts the cellular metabolism to the pentose phosphate pathway, decreasing lactate levels in pancreatic ductal adenocarcinoma. These metabolic pattern changes can promote autophagy and fatty acid oxidation, further reducing cancer cell growth^43^. ENO1 is involved in cell invasion and metastasis by promoting plasminogen activation into plasmin, which is a universal serine protease involved in ECM degradation^44^. Zhao et al. reported that high *ENO1* expression could serve as a biomarker for unfavorable prognosis in EC^45^. Upregulated *ENO1* expression translates into enhanced glycolysis intracellularly and increased ECM degradation and invasiveness extracellularly^44^.

PKM encodes a glycolytic enzyme that catalyzes the transfer of a phosphoryl group from phosphoenolpyruvate to adenosine diphosphate, thus generating ATP. *PKM* encodes two splice variants. Whilst *PKM1* is more efficient at producing pyruvate, the majority of proliferating cells and essentially all cancer cells express primarily the *PKM2* variant^46^. The increase in *PKM2* expression in metastatic cancer and its association with drug resistance in various malignancies have been demonstrated^47^. Besides its crucial role in metabolic reprogramming, PKM also serves as a cytosolic thyroid hormone receptor and epigenetic regulator of gene transcription in tumor cells^48^. Upregulated *PKM* expression results in a substantial modulation of metabolic fluxes, effective provision of various building blocks for cellular metabolism, and translation regulation. Moreover, regulatory properties of PKM2 may provide additional benefits to cancer cells by allowing them to better withstand oxidative stress^49,50^.

Nuclear receptor subfamily 2 group F member 1 (*NR2F1*) has been shown to modulate gene expression during cancer development and growth. Specifically, Gao et al. showed that *NR2F1* silencing stimulated cancer cell growth in vitro and suggested a role for the protein as a barrier to dissemination of early-evolved cancer cells^51^. NR2F1 is also implicated in hypoxia-driven glycolysis and migration of cancer cells^52^. Our results support the valuable role of *NR2F1* in tumor suppression. Conceivably, maintaining the *NR2F1* signal could reduce early dissemination of cancer cells and may represent a potential therapeutic option in EC.

N-acetylated alpha-linked acidic dipeptidase 2 (*NAALAD2*) encodes a transmembrane enzyme that cleaves neurotransmitters N-acetyl-L-aspartate-L-glutamate and beta-citryl-L-glutamate^53,54^. Since it releases C-terminal glutamyl residues, NAALAD2 could be implicated in reactive oxygen species-scavenging activity regulation and thus modulation of oxidative stress^55^.

Together, all the genes found in this study were shown to be implicated in cancer development and progression. Clearly, increased amino acid uptake, hypoxia-driven glycolysis, and increased ECM degradation and invasiveness create the principal metabolic pathways in EC.

Moreover, the screened genes were properly validated in clinical samples using qRT-PCR (Fig. 4A–K). The NanoString platform provided several key advantages, including sensitivity and robustness for analyses of FFPE samples^56^, and a strong evidence of utility of the nCounter for gene expression analysis. The qRT-PCR assay remains the most commonly used approach for mRNA expression profiling due to its high sensitivity and specificity. For these reasons it was used for the NanoString data validation. Both NanoString (which counts single molecules with no amplification step) and PCR (which utilizes an amplification method) provided similar expression patterns.

The identification of the key genes and biological pathways regulating tumor metabolism using different bioinformatic tools is crucial to discover molecular mechanisms underlying cancer development and progression. Our findings report metabolic pathways that are critical for EC cells and provide potential clinical targets for further investigation. GO enrichment analysis revealed that among the discovered DEGs were those involved in ‘neutral amino acid transmembrane transporter activity’, ‘regulation of plasminogen activation’, and ‘regulation of sulfur metabolic process’. Moreover, KEGG pathway analysis suggested that these DEGs were enriched in ‘CCM in cancer’ which includes glycolysis, the pentose phosphate pathway, and the tricarboxylic acid cycle (Figs. 1C and 2). CCM has been implicated in transport and oxidation of main carbon sources inside the cancer cell. EC cells coordinate the CCM pathways to balance the proliferative demands for the generation of energy and building blocks^57^. This evidence illustrates the strong relations between the DEGs and EC progression and supports the use of metabolic drug combination in EC therapy.

Further, in this study we undertook a search for a classifier for EC, somewhat in vain. Even if our metabolic investigation explored 768 genes, this number may have been too limited to construct a good classifier. Alternatively, these genes may be too functionally related. From our study, metabolism-related genes that may be useful classifiers in EC diagnosis have been proposed in Tables 1 and 2. In spite of their promising diagnostic value, additional analyses including genes not related to metabolism may be useful to create such a panel.

Drug repurposing enables the identification of compounds for use as treatments in diseases other than those for which they were originally developed. In this study, sapanisertib, PHA-793887, ciclopirox, sulfasalazine, and cytarabine were identified to target EC metabolism based on the transcriptomic signature (Table 3). Sapanisertib (also known as AK-228, MLN0128, INK128) demonstrated a manageable safety profile in phase-I clinical trials, with preliminary antitumor activity observed in renal cancer and EC. It is being developed both as monotherapy and in combination with other agents, such as paclitaxel, for the treatment of advanced solid tumors^58–60^. PHA-793887 belongs to the chemical class of pyrolopyrazole derivatives and is structurally different from the other CDK inhibitors. It is a potent inhibitor of multiple cyclin-dependent kinases, such as CDK2, CDK5, and CDK7. Therefore, it is an excellent a priori drug candidate for cancer therapy due to its potential to restore cell cycle control. Unfortunately, phase I clinical trials of PHA-793887 revealed unexpected hepatotoxicity in patients with solid tumors, however this side effect may be related to the simultaneous involvement of herpes simplex virus and other large DNA viruses^61,62^. In turn, ciclopirox is a synthetic antifungal agent commonly used as an olamine salt and also displays promising anticancer activity in preclinical models of several cancers^63–66^. A phase I clinical trial has shown its safety and tolerability in leukemia patients^67^. Besides, sulfasalazine is an anti-inflammatory drug used to treat autoimmune diseases, including ulcerative colitis and Crohn’s disease. It has been extensively studied for cancer treatment^68^. As for cytarabine, a synthetic pyrimidine nucleoside analogue, it has been used for the treatment of leukemia and lymphoma. Yet, it has been hypothesized that cytarabine demonstrates no particular anti-tumor activity in solid tumors due to a different intracellular expression of cytarabine metabolism-related enzymes^69,70^. Cisplatin, a well-known anticancer drug that acts by crosslinking with the guanine bases in DNA double-helix strands, has been used as a positive control to compare the effects of the repurposed drugs with standard chemotherapeutics^71^. To date, no data on clinical outcomes regarding selected repurposed drugs in EC patients are available.

Subsequently, in vitro validation was performed with the use of three EC cell lines, where the Ishikawa cell line represents the histological grade 1 EC, HEC-1B is consistent with grade 2 EC, and KLE is classified as poorly differentiated grade 3 EC^72^. Based on the results of these in vitro studies it can be assumed that EC metabolism differs between distinct EC grades. All studied compounds confirmed their cytotoxic effects in these cells at a range of 10^−9^ M–10^−5^ M, confirming in silico drug repurposing analysis. All these drugs, except for cytarabine, also increased caspase 3/7 activity in Ishikawa and/or HEC-1B cells. In contrast, no drug was found to change caspase 3/7 activity in KLE cells. This observation may emphasize the fact that cytotoxicity and apoptosis are two distinct phenomena. Increased caspase 3/7 activity reflects the regulation of mitochondrial function in apoptosis. Moreover, 17β-estradiol increased caspase 3/7 activity and glutamine uptake in Ishikawa cells, showing that glutamine metabolism can be regulated in estrogen-dependent manner, however, detailed mechanisms need to be further investigated.

One growth advantage of anaerobic glycolysis for cancer cells is that lactate generation is a faster reaction than OXPHOS. In our study, sapanisertib, PHA-793887, and ciclopirox decreased extracellular lactate concentration in Ishikawa and HEC-1B cells. Other groups have shown that lactate accumulation, and, therefore, acidic microenvironment, is a major factor in cancer cell invasiveness and lymph node metastatic colonization^73,74^. These observations highlight the potential benefit of targeting cancer acidity and/or lactate accumulation in EC.

Decreased extracellular glutamine and increased extracellular glutamate concentration reflect a greater intracellular consumption of glutamine. This could be a compensation mechanism of the decreased glycolytic rate after sapanisertib treatment in both Ishikawa and HEC-1B cells. Therefore, combined treatment strategies targeting cancer metabolism along with other pathways should be considered as potential treatment options. We observed decreased glutamate concentrations in HEC-1B cell media after both sulfasalazine and cytarabine treatment. While these compounds are known inhibitors of SLC7A11, glutamate excretion helps the cell to increase the nucleotide synthesis rate to sustain growth and manage the oxidative stress^75^. Nilsson et al. demonstrated that partial blocking of glutamate excretion due to SLC7A11 inhibition reduces liver cancer cell growth in vitro^76^. Targeting cellular metabolism using the repurposed drugs has the promise to improve therapeutic approach. For example, a clinical trial indicated a reasonable safety and preliminary antitumor activity of sapanisertib in both patients with renal cancer and EC^59^. Moreover, PHA-793887 has been studied as an alternative therapeutic in castration-resistant prostate cancer^77^. In turn, ciclopirox was observed to induce cell apoptosis in colorectal, pancreatic, cervical, and non-small cell lung cancer in vitro^64,65^. All in all, metabolic drugs under study may be a promising option for EC treatment via pleiotropic mechanisms including metabolism-dependent and -independent pathways. Such repurposed drugs as sapanisertib and ciclopirox should be considered for further studies in all EC grades, whereas PHA-793887 could be useful for grade 1 and 2 EC. More research is needed to confirm these initial analyses.

There is a number of limitations of the present study. First, not for all of the discovered DEGs drug-gene interactions were found. Consequently, their potential in EC treatment was not thoroughly investigated. The modulation of the DEGs’ expression in in vitro models has not been performed. The nCounter^®^ Metabolic Pathways Panel includes 768 genes covering the core pathways and processes that define cellular metabolism; a larger set of studied genes could provide a more comprehensive view of the metabolic profile in EC. Although the results were validated using qRT-PCR and analysis software, independent validation of the findings is necessary. Replicating the results in different sample sets will enhance the robustness of our findings. Additional studies in animal models and patient samples are needed to fully validate the clinical relevance of the presented results.

In turn, let us underline the strengths of the study. Reference tissues were obtained from carefully selected control subjects with normal endometrial morphology, and not from normal tissue from cancer patients. Therefore, the metabolic expression pattern investigated in normal tissues was not affected by nearby neoplastic processes. A relatively extensive material was studied. Further, the applied bioinformatic analysis was quite exhaustive. Presently, associating DEGs found in this study with clinical outcomes such as overall survival, disease-free survival, and treatment response in EC are justified and could provide meaningful insights into their clinical relevance.

In conclusion, we defined metabolism-related gene signatures in EC. This analysis confirmed the principal involvement of ‘CCM in cancer’ in EC metabolism. Our transcriptomic characterization identified both a unique metabolic EC phenotype and drugs predicted to be efficient in reducing EC progression. Further functional studies are necessary to determine the biological effects of the proposed candidate drugs.

## Methods

### Study subjects

This was a controlled study with a prospective method on a first-come basis. It was conducted on postoperative material collected from 87 patients hospitalized at the Department of Gynecology and Gynecologic Oncology, Medical University of Białystok, Poland. All subjects were white Caucasians representing an otherwise unselected population. Exclusion criteria were similar to those previously described^78^: prior treatment with chemotherapy, radiotherapy, or hormone therapy; coexistence of synchronous gynecologic malignancy or malignancy other than genital organs; stage IA1 cervical cancer (minimal microscopic stromal invasion); absence in postoperative material of uterine malignancy from earlier dilation and curettage biopsy; adenocarcinoma in polypo; and endophytic growth of the tumor (chiefly into the myometrium). In other words, surgery was required to be the first modality of EC treatment and only exophytic neoplastic lesions growing clearly into the uterine cavity were sampled. EC was anatomopathologically confirmed in 57 women (50 endometrioid, 4 serous, and 3 clear cell tumors). The classical three-tier grading classification (G1–G3) was applied^79^. Tumor staging was done by the International Federation of Gynecology and Obstetrics (FIGO) 2021 classification^79^, and tumor histologic typing was by World Health Organization International Classification^80^. This study was conducted in accordance with the principles of the World Medical Association’s Declaration of Helsinki, the International Conference on Harmonisation Guideline for Good Clinical Practice, and applicable laws and regulations of Poland. The study protocol was approved in advance by the Bioethics Committee of the Medical University of Białystok (Approval Number: APK.002.107.2020) and written informed consent obtained from each participant.

The reference group consisted of 30 women in whom anatomopathological examination did not reveal any pathological changes in the uterus. The indications for hysterectomy in this group were: heavy uterine bleedings refractory to medical treatment (N = 16), high-grade squamous intraepithelial lesion of the uterine cervix (N = 9), uterine prolapse (N = 2), and heavy postmenopausal bleedings due to endometrial (N = 2) and cervical (N = 1) polyps (Table 4).

**Table 4:** Clinical and anatomopathological characteristics of the studied groups.

Characteristic	EC patients (N = 57)	Reference women (N = 30)	*P*-value^a^
Age (median (range))	65.97 (34.50–86.50)	56.71 (36.00–78.00)	NS
Body mass (kg) (median (range))	81.66 (54.00–138.00)	72.31 (48.00–108.00)	< 0.05
Menopausal status (N (%))			< 0.001
Premenopausal	4 (4.50%)	10 (33.30%)	
Postmenopausal	53 (95.50%)	20 (67.70%)	
FIGO stage (N (%))			
I	39 (68.00%)		
II	5 (9.00%)		
III	12 (21.00%)		
IV	1 (2.00%)		
Cancer grade (N; %)			
1	18 (32.00%)		
2	25 (43.00%)		
3	14 (25.00%)		
Histology (N (%))			
Endometrioid	50 (88.00%)		
Non-endometrioid	7 (12.00%)		

^a^*P* values are shown for the χ2 test (categorical variables) and Mann–Whitney U test (continuous variables); FIGO, The International Federation of Gynecology and Obstetrics; Grading: 1–5% or less of tumor tissue is solid tumor growth, cancer cells are well-differentiated; 2–6–50% of tissue is solid tumor growth, cancer cells are moderately differentiated; 3–more than 50% of tissue is solid tumor growth, cancer cells are poorly differentiated; NS, no significance.

### RNA extraction

Total RNA was extracted from 3 consecutive 8 μm-thick FFPE sections. The tissue fragments were deparaffinized by incubation with Deparaffinization Solution (Qiagen, Hilden, Germany). For gene expression analysis, total RNA was extracted from deparaffinized tissue by proteinase K digestion and column chromatography using the RNeasy FFPE extraction kit (Qiagen) in accordance with the manufacturer's protocol. The RNA concentration was assessed by Qubit (Invitrogen, Carlsbad, CA, USA).

### nCounter gene expression assay

NanoString (NanoString Technologies, Seattle, WA, USA) is a molecular profiling technology that can generate accurate transcriptomic information from small amounts of preserved tissues. Overall, 768 genes involved in biosynthesis and anabolic pathways, nutrient capture and catabolic pathways, cell stress, metabolic signaling, and transcriptional regulation were studied. Briefly, mRNA samples were prepared by an overnight hybridization (65 °C) to nCounter Reporter and Capture probes. Samples were then placed into the nCounter Prep Station for automated sample purification and subsequent reporter capture. Each sample was automatically scanned on the nCounter Digital Analyzer for data collection. Reading with 555 field-of-views was used in the study samples. The NanoString data were deposited in the Gene Expression Omnibus (GEO) database (Accession Number: GSE196033).

### Validation of the NanoString results by quantitative real-time polymerase chain reaction

RNA was reverse transcribed using a High Capacity cDNA kit (Applied Biosystems, Foster City, CA, USA) according to the manufacturer's protocol on a BioRad C1000 thermal cycler (Mississauga, ON, Canada). Quantitative RT-PCR assays were run in triplicates on a LightCycler 480 Real-Time PCR System (Roche, Basel, Switzerland) and DEGs were quantified using KiCqStart SYBR Green qPCR ReadyMix and predesigned primers from Sigma-Aldrich (Saint Louis, MO, USA). Expression levels of investigated genes were normalized to geometric mean of the peptidylprolyl isomerase A (*PPIA*) and beta-actin (*ACTB*) housekeeping genes^81^ according with the Minimum Information for Publication of Quantitative RT-PCR Experiments (or MIQE) guidelines^82^. Gene expression was calculated using the qBase MSExcel VBA for relative quantification using the efficiency of gene-specific amplification.

### Data analysis

nSolver 4.0 Analysis software (NanoString) was used for data analysis, including normalization using housekeeping genes. The p-values were adjusted using the false discovery rate (FDR) of ≤ 0.05. The threshold value for significance of |FC| of ≥ 1.5 was applied to define DEGs. Statistical analyses were performed with GraphPad PRISM (v.9.1.1; GraphPad Software, San Diego, CA, USA). Preliminary statistical analysis (Shapiro–Wilk test) revealed that the studied parameters did not follow a normal distribution. Consequently, nonparametric Mann–Whitney U-test and χ^2^ test (for categorical variables) were used. Gene expression data for signature validation were obtained from the Genevestigator database (v.9.10.0; Nebion AG, Zurich, Switzerland), a public transcriptomic dataset of microarray data^83^. Data selection was performed using ‘cell lines’ type of condition and ‘endometrium’ from ‘neoplastic cell lines of the reproductive system’ was selected to obtain the data. Gene expression values were displayed in log2 scale. The DEG expression profile of non-treated human EC cell lines (AN3-CA, COLO 684, EFE-184, EN, HEC-1A, HEC-1B, Ishikawa, JHUEM-1, JHUEM-2, JHUEM-3, JHUEM-7, KLE, MFE-280, MFE-296, MFE-319, RL95-2, SNU-685, SNU-1077, TEN) was included. ECC-1 cell line has been excluded as it has been suggested to be a derivative of Ishikawa 3-H-12 or MCF-7 cells due to the contamination^84^.

Furthermore, an AUC for each DEG was calculated to evaluate the diagnostic value of the DEGs as candidate EC biomarkers. The analysis was performed using R software (v.4.0.3; R Core Team, 2020. R: A language and environment for statistical computing. R Foundation for Statistical Computing, Vienna, Austria; https://www.R-project.org/; package caTools)^85^. The AUCs and confidence intervals were calculated using the pROC^86^ and dplyr packages^87^.

### Gene ontology and pathway enrichment analysis of differentially expressed genes

To examine the functions of the identified DEGs, GO analysis and functional annotation clustering were carried out using the following four online databases: Gene Ontology enrichment analysis and visualization tool (GOrilla; http://cbl-gorilla.cs.technion.ac.il/), DAVID (Gene Ontology and KEGG Enrichment Analysis; https://david.ncifcrf.gov/), g:Profiler (https://biit.cs.ut.ee/gprofiler/gos), and Metascape (https://metascape.org). GO analysis allows for associating a given gene list with specific functional annotations, which are further divided into functional clusters listed according to their enrichment *p*-value^88^. KEGG pathway visualization^89^ was adapted using KeggParser plugin based on Cytoscape App (v.3.9.1; National Institute of General Medical Sciences, Bethesda, MD, USA; http://cytoscape.org/). To identify highly connected hub genes in PPI, STRING was applied at the interaction score > 0.4 (https://string-db.org).

### Modeling

An attempt was undertaken to construct a model based on a combination of biomarkers which would be a better classifier for EC. Features selected to be included into the investigated combinations were assessed by Weka software (v.3.8.6; Hamilton, New Zealand). For the comparisons (EC vs control), the feature selection chose two specific genes. Models based on logistic regression, naive Bayes and tree-based J48 algorithms were calculated. For each combination, confusion matrices were prepared to evaluate the model. Since the sample size was limited, the method ‘Leave One Out Cross-Validation’ was applied to control for the error.

### Drug repurposing

The Drug–Gene Interaction Database (DGIdb v.4.2.0; www.dgidb.org), Drug Signatures Database (DSigDB v.1.0; http://dsigdb.tanlab.org/DSigDBv1.0/), Drugbank (v.5.1.8; www.drugbank.ca), and Therapeutic Target Database (TTD v.7.1.01; http://db.idrblab.net/ttd/) were employed for drug repurposing. The candidate drugs for EC treatment were screened based on the above-identified DEGs only from compounds approved or investigational by the U.S. Food and Drug Administration (Silver Springs, MD, USA).

### Cell culture

Three EC cell lines: Ishikawa, HEC-1B, and KLE were selected to in vitro validate the drug repurposing results as they represent all three histological grades of EC according to the FIGO staging and differ in estrogen and progesterone expression status. Ishikawa cell line was purchased from Sigma-Aldrich (St. Louis, MO, USA) and HEC-1B and KLE were purchased from ATCC (American Type Culture Collection, Manassas, VA, USA). Ishikawa cell line was cultured in Minimum Essential Medium (MEM) supplemented with 5% fetal bovine serum (FBS, ATCC), HEC-1B was cultured in Eagle's Minimum Essential Medium (EMEM) supplemented with 10% FBS (ATCC), and KLE was cultured in Dulbecco’s Modified Eagle Medium/Nutrient Mixture F-12 (DMEM/F12) supplemented with 10% FBS (ATCC) in a 75 cm^2^ tissue culture flasks (Sarstedt, Nümbrecht, Germany) at 37 °C in a humidified atmosphere in the presence of 5% CO_2_. Cell culture was carried out with the substrate changed every two days until reaching 80–90% confluence. For experiments cells were trypsinized (0.25%, Gibco, Paisley, UK) and plated in medium with 0.5% charcoal-stripped FBS (Sigma-Aldrich) into white 96-well tissue culture plates (1 × 10^4^/well) (Corning Costar, Corning, NY, USA). The cell concentration and viability were determined using Countess device (Invitrogen, Thermo Fisher Scientific, Waltham, MA, USA). Cells were treated with drugs at various concentrations for 48 h. Following treatment, cell viability was evaluated using bioluminescent CellTiter-Glo^®^ 2.0 Cell Viability Assay (Promega, Madison, WI, USA) using GloMax^®^ Luminometer (Promega) in accordance with the manufacturer’s protocol. Likewise, caspase 3/7 activity was evaluated in cells using Caspase–Glo^®^ 3/7 Assay according to manufacturer’s instruction (Promega) using GloMax^®^ Luminometer (Promega).

Glucose, lactate, glutamine, and glutamate concentrations were evaluated in cell medium using bioluminescent assays (Glucose-GloTM Assay, Lactate-GloTM Assay, and Glutamine/Glutamate-GloTM Assay, respectively, all from Promega). Luminescence was measured using GloMax^®^ Luminometer (Promega) according to manufacturer’s instruction. Relative cell viability and metabolite concentrations were expressed as % change of treated cells vs. vehicle treated cells. Data are shown as the mean ± SEM of three independent experiments run in triplicates.

### Drugs

Sapanisertib (Selleckchem, Munich, Germany), PHA-793887 (Selleckchem), ciclopirox (Sigma-Aldrich), sulfasalazine (Sigma-Aldrich), cytarabine (Sigma-Aldrich), and 17β-estradiol (Sigma-Aldrich) were dissolved in dimethyl sulfoxide (DMSO) purchased from Sigma-Aldrich. The final concentration of DMSO never exceeded 0.1% and had no significant effect on cell viability. Cisplatin (Sigma-Aldrich) was solubilized in warm phosphate buffer saline (PBS, GIBCO, ThermoFisher, Cleveland, OH, USA). Therefore, the medium containing PBS was used as a control solely in case of cisplatin. After fasting for 24 h, the cells (1 × 10^4^/well) were washed twice with PBS, then were treated for 48 h with varying concentrations of sapanisertib, PHA-793887, ciclopirox, sulfasalazine, cytarabine, 17β-estradiol, and cisplatin (10^−9^ M–10^−5^ M). Based on literature review and preliminary viability assays (Fig. S3), specific concentrations were established for sapanisertib (10^−7^ M); PHA-793887 (10^−6^ M); ciclopirox (10^−6^ M); sulfasalazine (10^−6^ M); cytarabine (10^−5^ M); 17β-estradiol (10^−5^ M); and cisplatin (10^−5^ M), which were used for further studies.

### Ethics, consent, and permissions

A written informed consent was obtained from each participant.

### Supplementary Information

Supplementary Information.

## Data Availability

The dataset reported in this paper is available at GEO under the Accession Number GSE196033.
