# Supplementary material for: Identification and subsequent validation of transcriptomic signature associated with metabolic status in endometrial cancer
Source: Sci Rep. 2023 Aug 23;13:13763. doi: 10.1038/s41598-023-40994-w (PMC10447446; doi:10.1038/s41598-023-40994-w)
Supplement: Supplementary file 1 — Supplementary Information. [file 41598_2023_40994_MOESM1_ESM.docx]

**Supplementary Information for**

**Identification and subsequent validation of transcriptomic signature associated with metabolic status in endometrial cancer**

Iwona Sidorkiewicz, Maciej Jóźwik, Angelika Buczyńska, Anna Erol, Marcin Jóźwik, Marcin Moniuszko, Katarzyna Jarząbek, Magdalena Niemira, and Adam Krętowski

Table S1. DEGs with significantly different expressions between groups (FDR ≤ 0.05; |FC| ≥ 1.5).

| **Comparison** | **Gene** | **Fold change** | **p-value** | **FDR** |
| --- | --- | --- | --- | --- |
| Figo Grade 1 (N=18) vs reference group (N=30) | SLC7A5 | 2.28 | 0.000087 | 0.02 |
|  | RUNX1 | 2.25 | 0.000148 | 0.03 |
| Figo Grade 2 (N=25) vs reference group (N=30) | ACACB | -2.6 | 0.0000168 | 0.00 |
|  | AKT3 | -4.66 | 0.00000094 | 0.00 |
|  | ALDOA | 5.59 | 0.00000126 | 0.00 |
|  | BHMT2 | -2.32 | 0.00001684 | 0.00 |
|  | ENO1 | 7.54 | 0.00000011 | 0.00 |
|  | GAPDH | 8.45 | 0.00000499 | 0.00 |
|  | LDHA | 7.53 | 0.00000167 | 0.00 |
|  | MAP3K12 | -2.66 | 0.00000227 | 0.00 |
|  | PGK1 | 7.79 | 0.00000052 | 0.00 |
|  | PKM | 9.13 | 0.00000006 | 0.00 |
|  | AP2S1 | 3.43 | 0.00004207 | 0.01 |
|  | CYP1B1 | -2.87 | 0.00004197 | 0.01 |
|  | HAAO | -1.99 | 0.0000392 | 0.01 |
|  | LEPR | -4.1 | 0.00006778 | 0.01 |
|  | NPR2 | -2.42 | 0.0000921 | 0.01 |
|  | SLC16A7 | -2.09 | 0.00005426 | 0.01 |
|  | GNLY | -3.32 | 0.00015356 | 0.02 |
|  | NAALAD2 | -2.02 | 0.00015734 | 0.02 |
|  | PDGFRB | -7.77 | 0.00022282 | 0.02 |
|  | SLC16A1 | -2.34 | 0.00015283 | 0.02 |
|  | SLC16A2 | -2.48 | 0.00014072 | 0.02 |
|  | ATP6V1F | 2.65 | 0.00028457 | 0.03 |
|  | CD36 | -1.96 | 0.00033458 | 0.03 |
|  | AOC3 | -2.1 | 0.00041068 | 0.04 |
|  | LAMA4 | -6.47 | 0.00041359 | 0.04 |
|  | ADH1B | -3.41 | 0.00050252 | 0.05 |
|  | NPR1 | -1.78 | 0.00047277 | 0.05 |
|  | WNT2 | -5.53 | 0.00053044 | 0.05 |
| Figo Grade 3 (N=14) vs reference group (N=30) | BHMT2 | -2.16 | 0.00007287 | 0.01 |
|  | GAPDH | 5.58 | 0.00002765 | 0.01 |
|  | WNT2 | -5.04 | 0.00004009 | 0.01 |
|  | NR2F1 | -3.71 | 0.00013465 | 0.02 |
|  | NAALAD2 | -2.02 | 0.00015734 | 0.03 |
|  | AKT3 | -3.79 | 0.00022066 | 0.04 |
|  | HAAO | -1.86 | 0.00026288 | 0.04 |
|  | COL6A1 | -11.15 | 0.00031271 | 0.05 |
|  | NPR2 | -2,28 | 0.00035483 | 0.05 |
| Figo Grade 2 (N=25) vs Grade 1 (N=18) | SLC16A1 | -2.33 | 0.00012025 | 0.01 |
|  | AKT3 | -1.93 | 0.00048777 | 0.03 |
|  | ACACB | -1.49 | 0.00077143 | 0.05 |


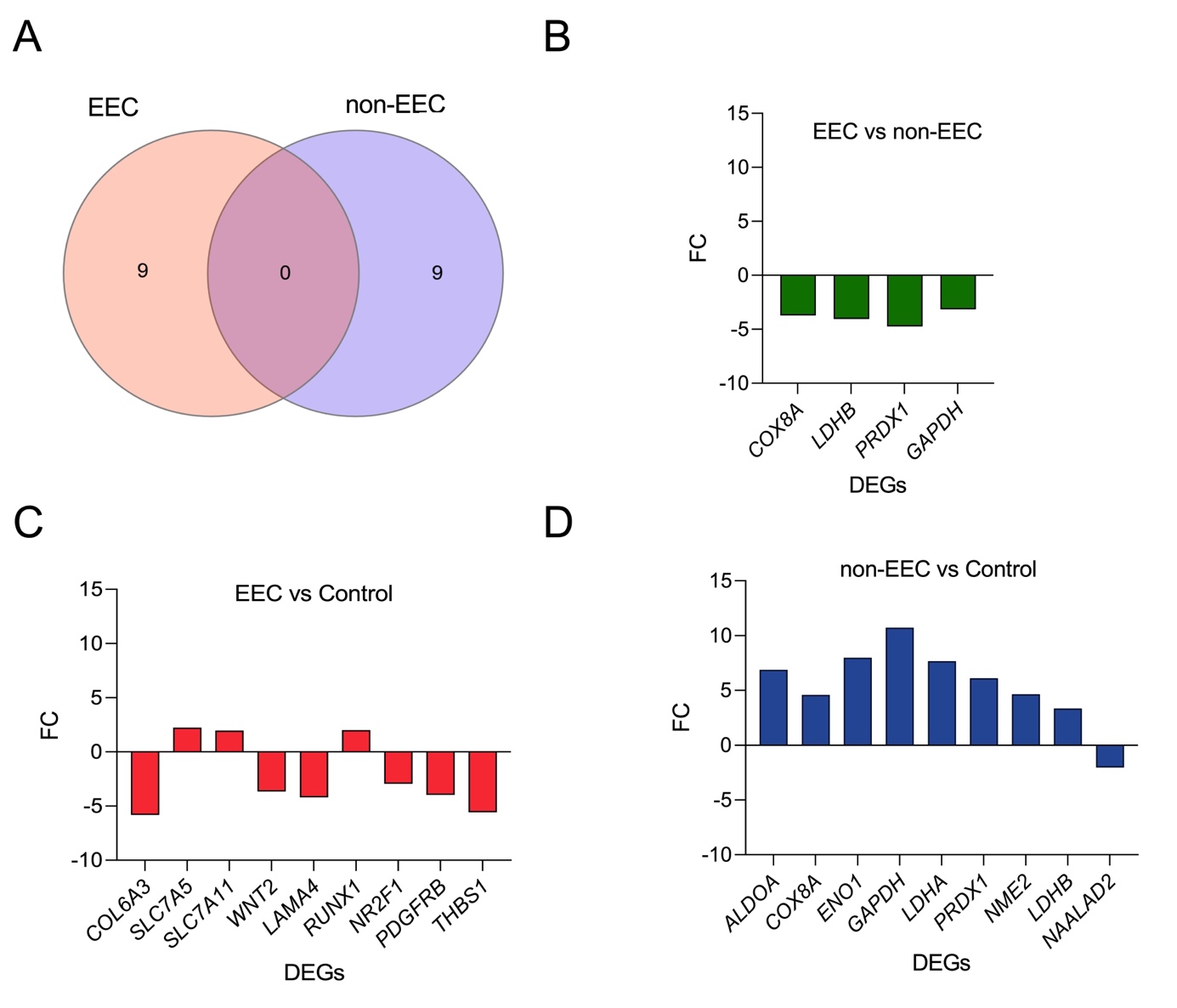
**Figure S1. (A**) Venn diagram showing the number of DEGs (FDR ≤ 0.05; |FC| ≥ 1.5) in EEC vs normal tissue, non-EEC vs normal tissue, and the number of overlapping DEGs between both subtypes of EC. (**B**) DEGs with significantly different expressions between EEC and non-EEC (FDR ≤ 0.05; |FC| ≥ 1.5). (**C**) DEGs with significantly different expressions between EEC and normal tissue (FDR ≤ 0.05; |FC| ≥ 1.5). (**D**) DEGs with significantly different expressions between non- EEC and normal tissue (FDR ≤ 0.05; |FC| ≥ 1.5). DEGs, differentially expressed genes; EEC, endometrioid EC; FC, fold change; FDR, false discovery rate.


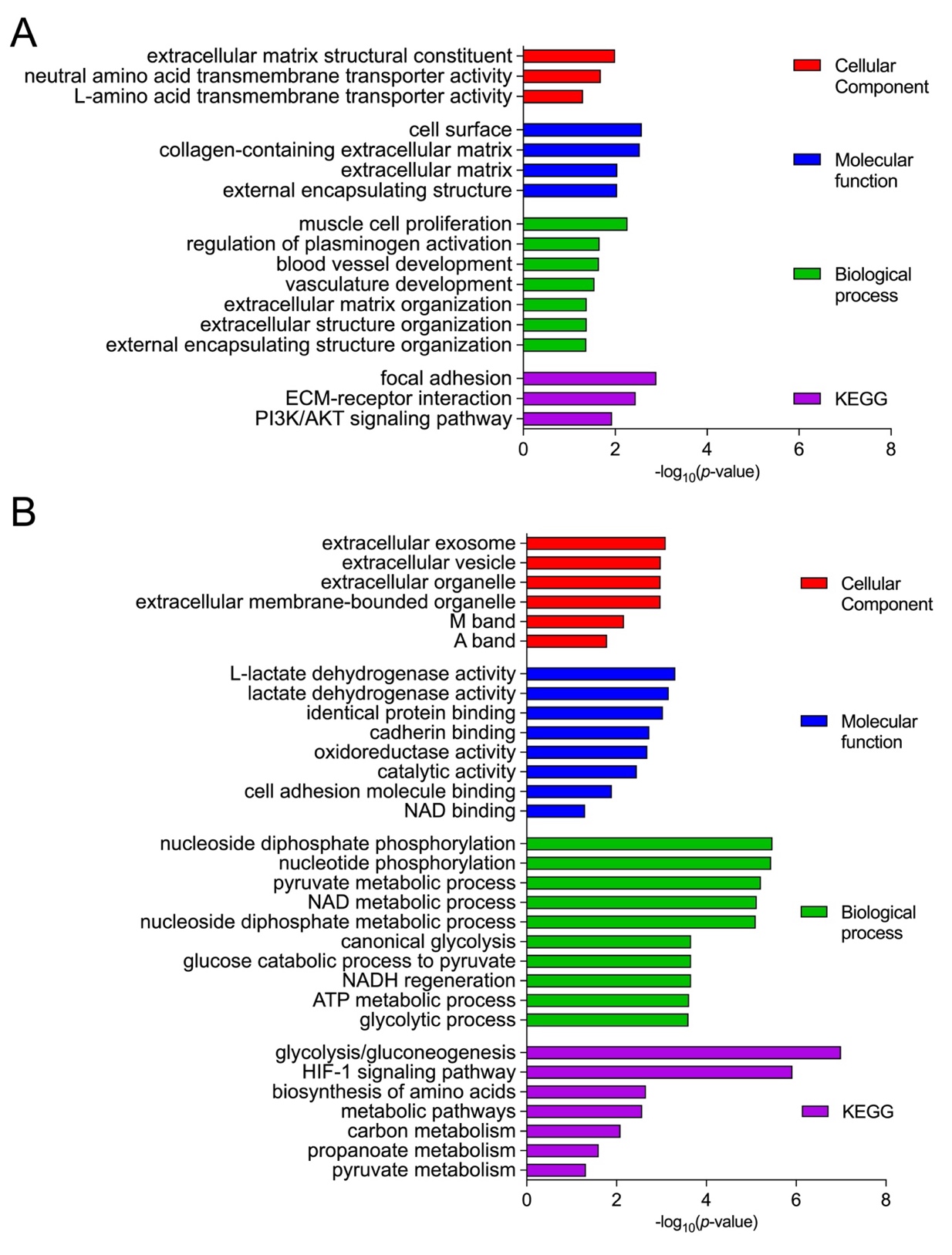


**Figure S2.** (**A**) GO enrichment analysis. Most significantly enriched GO (-log_10_(p-value)) categories of the DEGs in EEC vs normal tissue in the designated hallmark headings: cellular components, molecular function, biological process, and KEGG enrichment. (**B**) GO enrichment analysis. Most significantly enriched GO (-log_10_(p-value)) terms of the DEGs in non-EEC vs normal tissue in the designated hallmark headings: cellular components, molecular function, biological process, and KEGG enrichment.

**Differential Gene Expression in Endometrioid and Non-Endometrioid Endometrial Cancer.**

Differential expression analysis to identify the transcriptomic profile associated with the histologic subtypes of EC was performed to detect DEGs in endometrioid EC (EEC) and non-EEC tissues. Interestingly, there was no overlap between EEC and non-EEC gene signatures when compared with controls (**Figure S1A**). Expression of cytochrome C oxidase subunit 8A (*COX8A*), lactate dehydrogenase B (*LDHB*), peroxiredoxin 1 (*PRDX1*), and glyceraldehyde 3-phosphate dehydrogenase (*GAPDH*) was decreased in EEC when compared with non-EEC based on an FDR threshold of ≤ 0.05 and |FC| of ≥ 1.5 (**Figure S1B**). The list of DEGs in EEC and non-EEC compared with normal endometrial tissue is provided in Figures S1C and S1E. GO analysis identified a predominant involvement of extracellular matrix (ECM) pathways in EEC and glycolytic processes in non-EEC (**Figures S2A**and **S2B**).


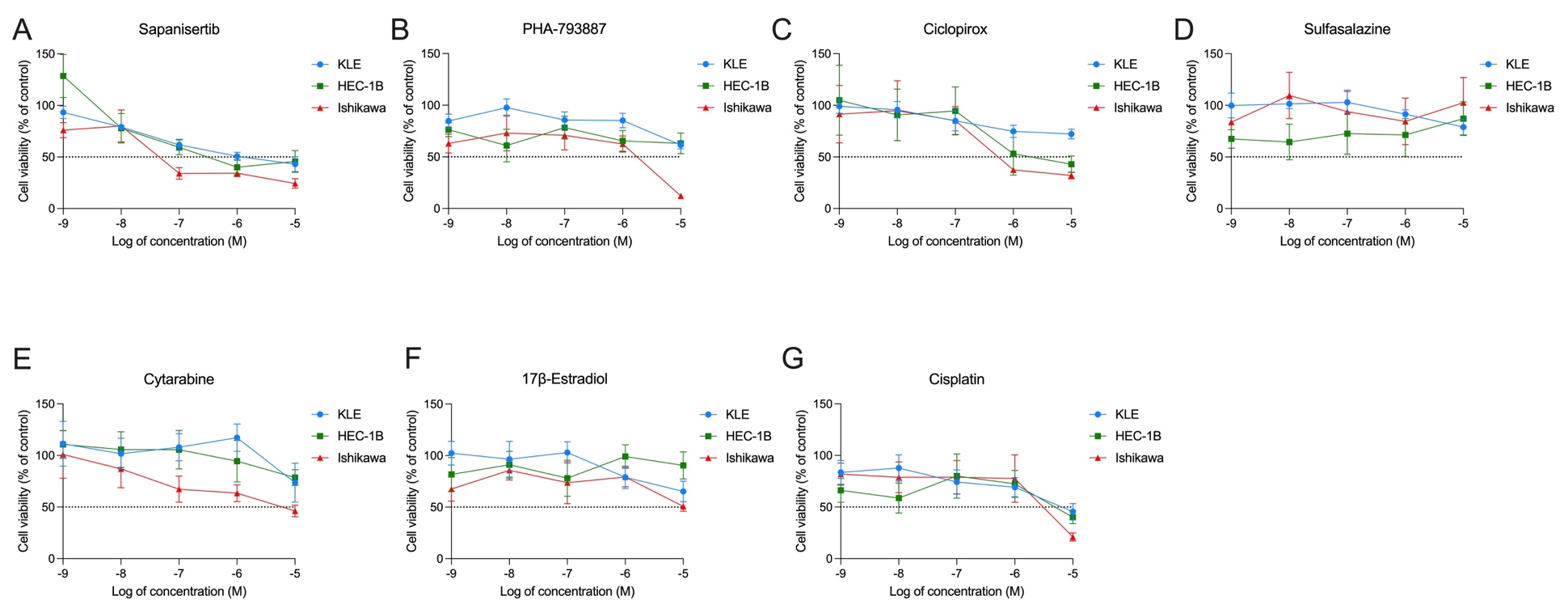


**Figure S3.** Effects of predicted drugs on cell viability in three endometrial cancer cell lines: Ishikawa, HEC-1B, and KLE. Cells were treated for 48h with (**A)** sapanisertib, (**B**) PHA-793887, (**C**) ciclopirox (**D**) sulfasalazine, (**E**) cytarabine, (**F**) 17β-estradiol, and (**G**) cisplatin at concentrations 10^−9^ M - 10^−5^ M. Each data point was normalized to the control (dimethyl sulfoxide, or phosphate-buffered saline (PBS) solely in case of cisplatin) and represents the mean ± SEM from three independent experiments. Cell viability was assessed by CellTiter-Glo^®^ 2.0 Cell Viability Assay.
